# Anterior Cruciate Ligament Rupture in Skeletally Immature Patients

**DOI:** 10.5435/JAAOSGlobal-D-21-00166

**Published:** 2022-05-17

**Authors:** Benjamín Cancino, Carlos Muñoz, María Jesús Tuca, Estefanía A. M. Birrer, Matías F. Sepúlveda

**Affiliations:** From the Universidad Austral de Chile, Valdivia, Chile (Dr. Cancino, Dr. Muñoz, Dr. Birrer, and Dr. Sepúlveda); the Clínica Alemana, Santiago, Chile (Dr. Tuca); the Universidad del Desarrollo, Santiago, Chile (Dr. Tuca); the Hospital Clínico Mutual de Seguridad, Santiago, Chile (Dr. Tuca); and Hospital Base de Valdivia, Valdivia, Chile (Dr. Birrer, and Dr. Sepúlveda).

## Abstract

In the past 20 years, sports injuries in pediatric and adolescent athletes have increased dramatically, with anterior cruciate ligament (ACL) injuries accounting for more than 25% of all knee injuries at this age. Diagnosis is based on detailed clinical history, physical examination, and imaging assessment, where magnetic resonance imaging plays a central role. The growing immature skeleton presents specific characteristics, which require unique methods for surgical reconstruction, ideally avoiding the physes or minimizing the risk of damaging them. Specific rehabilitation protocols are needed, and these patients face a higher risk of recurrent and contralateral ACL injury. Nonsurgical treatment or delayed reconstruction has been associated with persistent instability, activity modifications, worst functional outcomes, and increased risk of irreparable injuries to menisci and articular cartilage. Consequently, surgical stabilization is the preferred treatment for most patients, despite the eventual risk of angular deformities or limb-length discrepancies due to iatrogenic physeal injury. A variety of surgical techniques have been described, depending on the skeletal maturity and growth remaining. Targeted prevention programs play a key role in reducing the risk of ACL injury, are easy to implement, and require no additional equipment. High-quality evidence supports its use in all pediatric athletes.

In the past 20 years, sports injuries in pediatric and adolescent athletes have increased dramatically.^1^ This new epidemic of sports-related injuries can be attributed to an increase in the number of athletes, better recognition of such injuries, early sports specialization, high level of competition, and high-intensity training during most of the year.^2-4^

Knee injuries account for approximately 50% to 60% of all sports-related surgical procedures in high school,^5,6^ and anterior cruciate ligament (ACL) injuries account for more than 25% of all knee injuries.^7^ The ACL injury rates vary by sport, sex of the athlete, level of competition, and type of exposure, with an overall rate of 6.5 injuries per 100,000 athletes among United States' school athletes.^8^

Skeletally immature patients require unique methods for surgical reconstruction, ideally avoiding the physes or minimizing the risk of damaging them.^9^ They also require different rehabilitation protocols owing to the variations in reconstruction methods, age, and grade of skeletal maturity.^10,11^ In addition, younger patients are generally more active than adults and have a higher risk of recurrent ACL injury.^12^ Patients younger than 21 years have a revision surgery rate that is 7.76 times higher, with an average graft rupture rate of 13% to 19%.^13-15^

Historically, the possible iatrogenic lesion of the physis has limited ACL reconstruction in skeletally immature patients because of the risk of angular deformities or length differences in the extremities.^10^ However, this paradigm has changed with the presentation of other complications in the natural history of this injury, such as persistent joint instability, limitations in physical activity, increased risk of irreparable injuries to the menisci and joint cartilage, and worst functional results that have been observed with late reconstruction and nonsurgical management of these patients.^16,17^

## Anatomy

**Figure 1:**
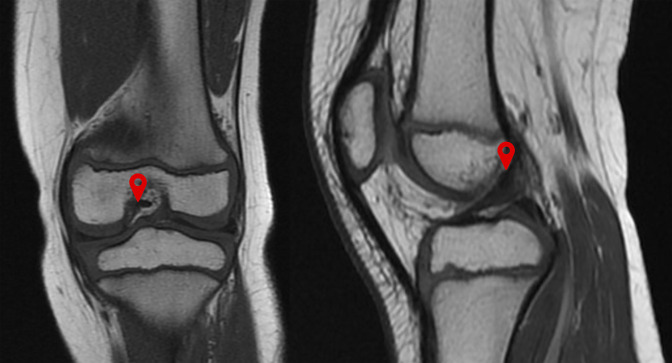
Coronal and sagittal T1-weighted MRIs showing the knee of a 6-year-old girl, marking the femoral insertion of the anterior cruciate ligament.

The distal femoral flat primary physis contributes to approximately 70% of the total femoral length and 37% of the total limb length during skeletal development, at an average growth rate of 10 mm per year. The distal femoral secondary ossification center enlarges globally from its own spherical secondary physis.^18^ The distance between the flat primary physis and the femoral origin of the ACL remains unchanged until skeletal maturity and averages approximately 3 mm^19,20^ (Figure 1).

The width of the femoral intercondylar notch continues to increase steadily through skeletal development until the age of 11 years in boys and girls, after which there is no notable increase in the width of the anterior portion of the notch.^21^ The lateral intercondylar ridge (resident's ridge) lies on the medial aspect of the lateral femoral condyle and marks the anterior edge of the ACL femoral footprint with the knee in 90° flexion.^22^ It is important to note that this landmark is used to determine the accurate position of the femoral tunnel during ACL reconstruction, considering that this may vary, and in fact, several studies have shown that this reference may be present less frequently in younger children (88% in 13- to 20-year-old patients versus 44% to 63% in 3- to 12-year-old patients).^20,22^

**Figure 2:**
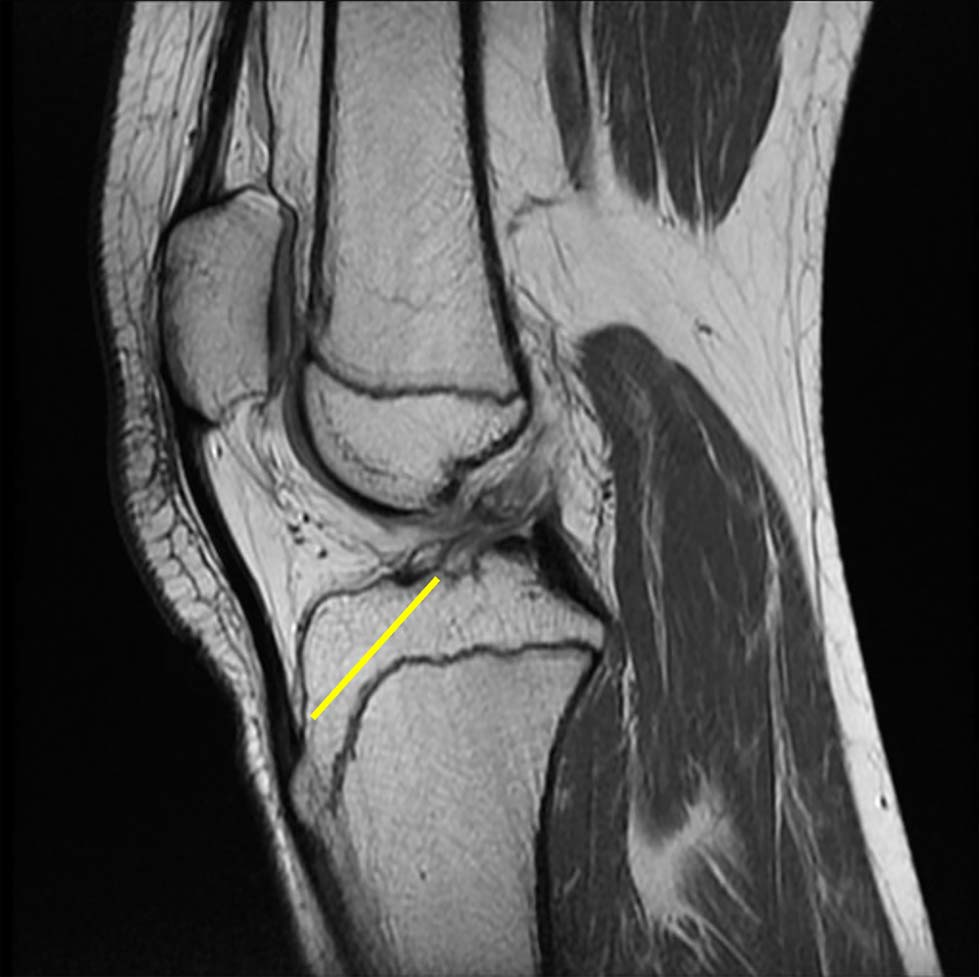
Sagittal T1-weighted MRI showing the knee of a 13-year-old boy, measuring of the maximum oblique intraepiphyseal depth.

The proximal tibial physis contributes approximately to 55% of the total tibial length and 25% of the total limb length during skeletal development, at a growth rate of 6.4 mm per year. An important anatomical parameter is the maximum oblique intraepiphyseal depth measured from the tibial insertion center of the ACL, moving anteriorly and distally, thus achieving the greatest oblique distance, avoiding the physis. This depth gradually increases with growth, from a mean value of >20 mm in preadolescents to approximately 30 mm in the adolescent group^22^ (Figure 2).

Among adolescents, the center of the tibial union of the ACL is around 50% of the AP distance of the tibia, regardless of age or sex.^23^ This is important information for the location of the tibial tunnel in transphyseal ACL reconstruction in patients with an immature skeleton, where the goal is to position the tunnel in the most central region of the tibial physis to reduce the risk of axial deformity or iatrogenic injury to perichondral structures.^24^ Because physeal arrest of the tibial physis is a rare complication, an anatomic tibial tunnel position for ACL reconstruction techniques should be the goal.

The ACL grows in length and diameter with age. Younger patients have a more oblique ACL than older patients. The growth model for ACL length shows three distinct phases: Patients aged between 1.5 and 5.75 years have an average growth of 2.25 mm per year, and those aged between 6 and 11.5 years have an average growth of 1.46 mm per year; growth begins to stabilize at 11.75 years and stops at 18.5 years. The growth model for the sagittal diameter of the ACL shows an average of 0.45 mm of growth per year between 1.5 and 14.5 years, after which the growth slows until it stops at 18.75 years. The coronal diameter of the ACL shows an average growth of 0.22 mm per year between 1.5 and 18.75 years, with complete growth at the age of 18.75 years.^21,25^

## Injury Risk Factors

Multiple risk factors associated with ACL rupture have been described, both in adult and pediatric populations, some without completely clear evidence. These risk factors are divided into nonmodifiable and modifiable risk factors.

### Nonmodifiable Risk Factors

Patella alta, a smaller notch width index, and a lower volume of the intercondylar notch (compared with those in adults) have been described as risk factors for ACL injury in skeletally immature patients.^26^ During puberty, rapid growth occurs in the femur and tibia, increasing the torque force on the knee and making muscle control more difficult, thus conferring a greater risk of ACL rupture during this period of development.^27^ In the adolescent population that participates in sports activities, the risk of ACL rupture is found to be 1.6 times higher in female than male subjects. The reason for this is not entirely clear but seems to be based on biomechanical and hormonal theories.^28^ This difference in the risk of ACL rupture is not observed in the prepubertal population.^27^ Another characteristic feature of the pediatric population (skeletally immature patients) is the presence of hypermobility, which is considered to markedly increase the risk of injury, especially in girls who play soccer.^29^

### Modifiable Factors

In this group of factors, the neuromuscular imbalance, typical of developing children, takes main importance. Within this concept, the presence of a dominant limb, quadriceps dominance, dynamic instability, and neuromuscular activation patterns are included.^30^ There is risk related to the type of footwear, especially those that provide greater torsional resistance to the ground. The type of soil also seems to play a role in the risk of injury, especially those with higher shoe-surface friction. Indoor sports would have a higher risk than a sport on grass. The weather also affects the interaction between the footwear and the surface. Studies have shown that ACL injuries are less common during the low water evaporation and high rainfall season in Australian football and during cold weather in American football. Finally, the type of sport to be played can be modified, thus reducing the risk of injury.^30,31^

## Diagnosis

The diagnosis of ACL lesions should include a detailed clinical history, physical examination, and imaging assessment. The patient usually presents with a painful effusion of the affected knee and avoids load bearing on the affected limb. Unlike adult patients, children often fail to provide a clear history regarding the mechanism of trauma. Overall, 65% of young athletes with hemarthrosis have an ACL rupture.^28,32,33^ Positive Lachman tests (sensitivity: ∼96%), anterior drawer, and pivot shift are usually present but are limited by pain and swelling in the acute phase. Pediatric patients show poor tolerance to these tests, thereby reducing the validity of the tests. The pivot shift can be done under sedation, reaching 98% positivity.^27,32^ Given the physiologically increased generalized joint laxity in the pediatric population, comparative testing of the contralateral limb is of utmost importance.^32^ We must also evaluate other underlying conditions in the patient, such as limb-length discrepancy or axis alteration.^32^

**Figure 3:**
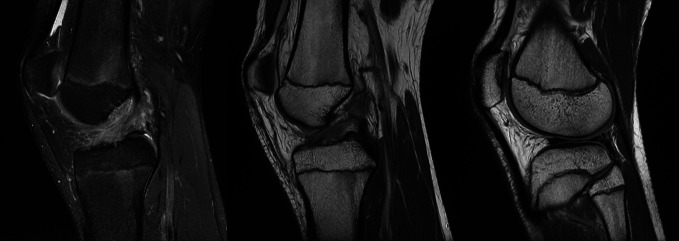
Sagittal T1-weighted and STIR-weighted MRIs showing the knee of a 15-year-old boy, with a complete anterior cruciate ligament rupture and associated meniscal tear.

AP, lateral, and intercondylar tunnel radiograph views are recommended to rule out trauma-related bone injuries. Among these, it is important to identify avulsion fractures of the tibial spines that correspond to an ACL rupture equivalent in the population with an immature skeleton.^34^ In all patients suspected of an ACL injury, a magnetic resonance image (MRI) of the knee should be obtained, which has a sensitivity of 95% and a specificity of 88% in children with ACL rupture (Figure 3). It also provides important information regarding commonly associated injuries, such as meniscal tears, chondral injuries, and other ligament injuries, which may have implications for the treatment of the patient.^28,32,33,35^

## Treatment

A discussion of the appropriate management of the injury and understanding the goals and expectations of the patients and parents are crucial for making the treatment decision.

Nonsurgical treatment of ACL injuries in pediatric patients may be part of a definitive strategy or a temporary span to gain skeletal and psychological maturity. Nevertheless, conservative treatment should be cautiously indicated because multiple studies show that nonsurgical strategies, or delayed surgical stabilization, associate with articular damage and sports dropout in pediatric patients. On the other hand, systematic reviews showed that early surgical stabilization reduces pathological laxity and improves return to activity in young athletes.^36^ Our recommendation is to indicate conservative treatment only to low-demand patients, patients willing to adopt activity modifications and restrictions, or patients with partial ACL injuries that do not present with subjective instability or knee failure in daily activities. Monitoring patients undergoing conservative treatment is crucial to detect persistent instability or signs of articular damage. An MRI follow-up is also advised to identify articular injuries or instability episodes.

Conservative treatment relies on proper and systematic rehabilitation exercises, which are critical in treating ACL injury. The same principle applies for rehabilitation, regardless of whether the patient has had an ACL reconstruction or has decided to undergo nonsurgical treatment. The bases of rehabilitation in children are based on the clinical experience and research done in adults, although it is not entirely clear whether these principles apply to children. Additional studies should be conducted to prospectively evaluate rehabilitation protocols and return-to-sport criteria for young athletes while keeping in mind both physical and psychosocial differences between children and adults.^37^

Rehabilitation must be comprehensive and individualized to the physiological and psychological maturity of the child to achieve successful results. Exercises that strengthen dynamic lower extremity alignment and biomechanically correct movement patterns should be emphasized. Rehabilitation programs should be designed to allow children to stay within their sports environment, such as participating in team training sessions. Parents or guardians must be active participants in daily rehabilitation. This may include assisting the child in technical and functional exercises during training with their equipment.^35,37^

Rehabilitation for a child with an ACL injury is organized into four phases, with an additional rehabilitation phase for those who have undergone ACL reconstruction. Specific clinical and functional milestones must be reached before advancing from one phase to the next one.^38^ Throughout the first two phases, the child must be protected from deceleration and rotation activities. The rehabilitation progression scheme through functional milestones is similar for ACL reconstruction and nonsurgical treatment. However, there are different expectations for progression and time to fully return to sports.^39^ Nonsurgical rehabilitation treatment should last at least 3 to 6 months.^40^ Postoperative rehabilitation should last a minimum of 9 months before the patient can fully participate in specific sports activities.^41^

The recommended goals for each phase of rehabilitation are as follows^35^:

The presurgical phase for patients scheduled to undergo ACL reconstruction include the following:

1. Active full extension and at least 120° flexion
2. Little or no joint effusion
3. Maintain extension with a standing support leg
4. Adolescents: 90% symmetry in leg strength

Phase I to phase II goals in patients with ACL reconstruction or nonsurgical treatment are as follows:

1. Active full extension and at least 120° flexion
2. Little or no joint effusion
3. Maintain extension with support on a standing limb

Phase II to phase III goals in patients with ACL reconstruction or nonsurgical treatment are as follows:

1. Full range of motion of the knee
2. 80% symmetry in extremities in single-leg jump tests, with adequate landing
3. Ability to run correctly for 10 minutes and without subsequent spillage
4. Adolescents: 80% symmetry in leg strength

Phase III to phase IV goals for patients with ACL reconstruction or nonsurgical treatment, sports participation criteria, and continued prevention of injuries are as follows:

Single-leg jump tests: >90% of the contralateral limb (with adequate quality of movement)
Gradual increase in specific sports training, without pain or joint effusion
Confidence in knee joint function
(4)Knowledge of positions with high risk of injury and ability to maintain low-risk positions in sports practice
Mentally prepared to return to sport
Adolescents: 90% symmetry in leg strength

Surgical decision on the treatment of ACL tears in patients with an immature skeleton is challenging and remains a controversial issue among surgeons. The most common indications for surgical stabilization are (1) failure of conservative treatment, when the patient, despite completing rehabilitation, maintains subjective instability and cannot return to their usual activities; (2) associated repairable injuries (meniscal and osteochondral); (3) multiligamentary injuries; and (4) a patient who is actively involved in sports who does not tolerate a modification of their activities.

Evidence indicates that to minimize the risks of physeal injury, it is of utmost importance to adequately determine the bone age and physiological maturation of the patient and choose the most appropriate technique for each patient according to their stage of development. The Radiographic Atlas of the Hand and Wrist published by Greulich and Pyle^42^ in 1959 has been the most used method by radiologists and orthopaedic surgeons for this task, but it presents difficulties at the time of its application, mainly because of the low intraobserver and interobserver agreement. The Shorthand Bone Age is proposed as an alternative, which despite its good intraobserver-interobserver relationship does not manage to overcome the Greulich and Pyle method but seems to be a useful tool for this group of patients.^43^ The simplified skeletal maturity scoring system by Sanders et al^44^ is widely used to assess the risk of scoliosis progression, proven to be a reliable predictor of the curve acceleration phase, but is not very popular for other skeletal segments. In addition, it is generally recommended to conduct a complementary evaluation with the Tanner and Whitehouse^45^ scale for an adequate correlation with patients' physiological development. However, some studies have shown that the Tanner staging done by orthopaedic surgeons is inaccurate and unreliable and may lead to erroneous decisions regarding the treatment, given that this is a maturity index and unsuited for patient's age estimations.^46^ Kelly and Dimeglio^47^ created tables where the estimate of residual growth of each segment is indicated according to the bone age and sex of the patients. Current studies recommend this method as being more reliable than the Tanner scale for decisions regarding treatment and prevention of complications.^48^

Various surgical techniques classified as “transphyseal” and “physeal-sparing” are described for managing these lesions. The literature describes certain surgical principles for ACL reconstruction that should be taken into consideration to reduce the risks of secondary alterations of the physis in patients with an immature skeleton.^24^ No technique has been shown to be superior, with all being viable alternatives, provided the principles are respected.

**Figure 4:**
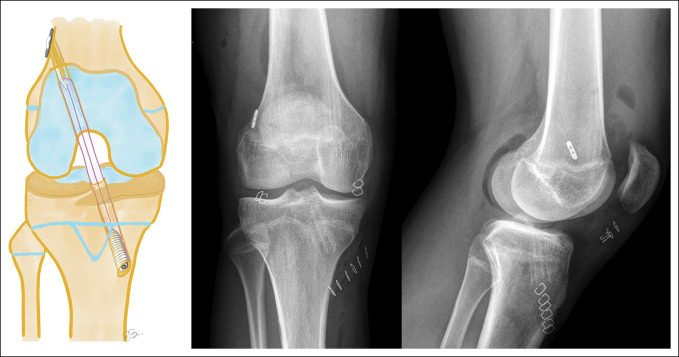
A schematic diagram showing the transphyseal reconstruction technique. AP and lateral postoperative radiographs showing the knee of a 14-year-old boy with a transphyseal anterior cruciate ligament reconstruction.

The “transphyseal technique” uses bone tunnels in both the tibia and the femur through the physis, highlighting that the femoral tunnel has a vertical central orientation and a small diameter to avoid physeal arrest (Figure 4). It uses a soft-tissue graft, reducing bone bar formation. Implant fixation elements should be placed away from the physis. The main advantage of this technique is that it resembles the one used for adult patients, and surgeons are therefore more familiar with it. The limitations are the nonanatomical position of the ligament, risk of rotational instability, smaller diameter graft, and potential physeal damage.^49^

**Figure 5:**
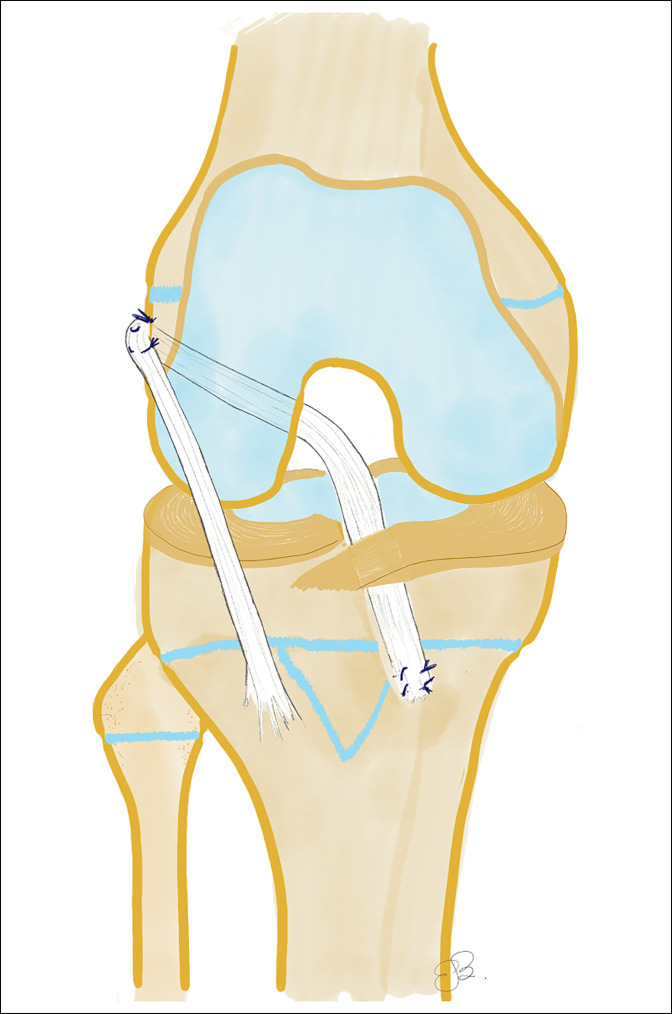
A schematic diagram showing the extraphyseal reconstruction technique. The iliotibial band graft is harvested free proximally and left attached to the Gerdy tubercle distally, then brought through the knee in the over-the-top position posteriorly, through the knee and under the intermeniscal ligament anteriorly.

The “physeal-sparing” techniques are divided into “extraphyseal” and “all-epiphyseal” types. The “extraphyseal” type corresponds to a combined intra-articular technique with an extra-articular reconstruction by using an autograft of the iliotibial band (ITB). The graft is first harvested from the ITB, detached from the proximal portion to preserve its distal insertion in the Gerdy tubercle. Arthroscopy identifies the over-the-top position in the femur and the over-the-front position below the intermeniscal ligament. The graft is then prepared and retrieved, passed through previously identified landmarks. Next, an incision is made in the medial proximal tibia at the insertion of the pes anserinus. Finally, an incision is made at the level of the periosteum, distal to the tibial physis, and the distal end is sutured to the periosteum (Figure 5). The main advantage of this technique is that it does not require bone tunnels and hence prevents physeal damage. The drawbacks are that it is not an anatomical technique, and biomechanical studies have shown that there is notable joint stiffness after reconstruction.^49^

**Figure 6:**
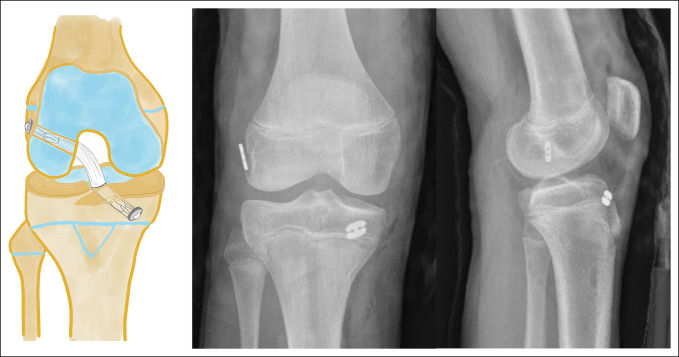
A schematic diagram showing the all-epiphyseal anatomical reconstruction technique. AP and lateral postoperative radiographs showing the knee of a 12-year-old boy with an all-epiphyseal anterior cruciate ligament reconstruction.

**Figure 7:**
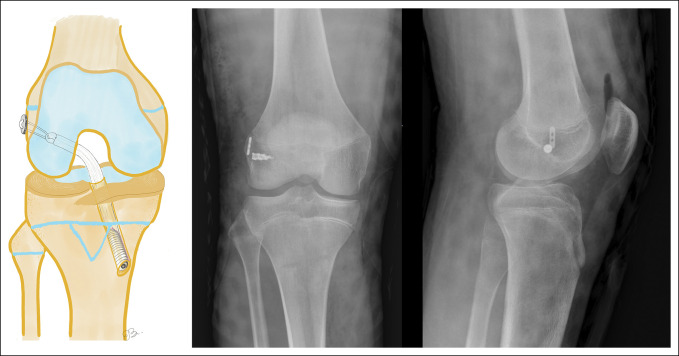
A schematic diagram showing the hybrid reconstruction technique, with an all-epiphyseal femoral tunnel and transphyseal tibial tunnel. AP and lateral postoperative radiographs showing the knee of a 13-year-old boy with this technique, associated with an anterolateral ligament reconstruction fixed with a suture anchor.

The “all-epiphyseal technique,” described by Anderson,^50^ includes bone tunnels completely contained within the epiphysis and guided under fluoroscopy in both views to specifically avoid the physis (Figure 6). The main advantage of this technique is that it respects the physis and positions the tunnels in an anatomical location.^51^ However, it is technically demanding and requires special instruments and fluoroscopic support during surgery.^49^ It is also not exempt from damaging the physis, given its proximity to it at the time of tunneling. Because of this, hybrid techniques have been frequently used, with an epiphyseal femoral tunnel avoiding the physis and a central transphyseal tibial tunnel (Figure 7).

Autograft is the preferred option for reconstruction techniques, but its main drawback is the morbidity of the donor site. The use of patellar tendon (BTTB) graft is limited in pediatric patients because it relies on bone-to-bone fixation, limits its use in transphyseal or physeal-sparing techniques, and the screw position can affect the physis. Moreover, patellar tendon graft does not allow for graft length adjustments, critical for physeal-sparing techniques, and is associated with anterior knee pain and reduced force in knee extension. Hamstring (HT) autografts generate flexion weakness with possible complications related to walking or running but allows adjustments to total graft length and the use of suspensory fixation systems. Furthermore, in the pediatric population, this option can lead to very small size grafts, limiting successful reconstruction.^52^ Quadriceps tendon (QT) is being increasingly considered for pediatric ACL reconstruction during the past few years. QT is a purely soft-tissue graft of predictable length and diameter, with strong tensile properties and a large cross-sectional area that avoids apophyseal injury in comparison with BPTB grafting, and has reduced donor-site morbidity in comparison with Bone-Patellar-Tendon-Bone (BPTB) or hamstring tendon (HT) grafts.^53,54^ Nevertheless, it is not exempt from risks.^55,56^ Shea et al^57^ published a cadaver study in pediatric specimens, describing the anatomy of the pediatric QT and decussation of the rectus tendon, to avoid the risk of delayed rectus tendon retraction and quadriceps weakness after QT graft harvest. Surgeons should be aware of the separation of the rectus tendon from the QT, and the coronal width variations in pediatric patients, to avoid inadvertent release of the rectus tendon from the rest of the QT complex when using this graft in skeletally immature patients. Recent studies have established QT as a reliable option for ACL reconstructions in adults because it shows similar graft survival, functional outcomes, and stability compared with BPTB and HT autografts, with a lesser donor-site morbidity.^58^ Gagliardi et al^59^ showed excellent stability and favorable patient-reported outcomes after a 2-year follow-up of 81 adolescent patients using QT graft for all-epiphyseal, transphyseal, or hybrid ACL reconstructions. So, despite there being still less reported evidence of QT versus HT or ITB in the pediatric population, findings so far make it a valid and rising option for pediatric ACL reconstructions. The technique choice will be the main determinant in autograft selection, with the hamstrings being most prevalent in the pediatric population, followed by the ITB graft.^60^

Allografts are an alternative to prevent donor-site morbidity, reduce surgical time, and better predict the diameter of the graft. However, they have shown a higher rate of rerupture, especially in young athletes; therefore, it is not recommended in this population.^61,62^

Another alternative for pediatric patients is the use of a hamstring graft from a living donor (one of the parents).^63,64^ This graft is advantageous in that it does not need to be frozen, irradiated, or pretreated; avoids complications of the donor site; promises a larger graft diameter; and has shown good survival and the absence of complications in a 5-year follow-up, especially in patients with Tanner stages 1 to 2 at the time of surgery.^52^

The size of the graft directly affects the risk of rerupture in the adult population, showing a greater risk of revision with grafts that have a diameter of <8 mm, especially in the population younger than 20 years.^65^ Cruz et al^66^ did not identify a statistically significant difference in the risk of rerupture according to the graft diameter in their pediatric population, highlighting that the diameter is important in relation to the height of the patient and that, theoretically, the shorter the patient, the smaller the diameter of the harvested autograft. Independent of this, it is also identified that autografts in general measure ≥8 mm.

One option for very small diameter grafts is to conduct allograft augmentation. Perkins et al^67^ demonstrated that reconstruction with allograft augmentation has a 2.6-fold increased risk of failure than reconstruction with an autograft. In addition, they showed a similar risk of failure in four-strand and five-strand grafts. Given this, techniques to triple the graft to obtain a larger diameter are preferred over the use of allograft in the pediatric population.

## Outcomes

ACL plays a vital role in knee stability. In general, conservative management has a lower rate of sports return and a greater association with injuries secondary to instability (mainly meniscal and chondral injuries) than the surgical group. A nonsurgical protocol could be a reasonable option for patients who could comply with strict activity modification.^37,68^ Moksnes^69^ studied the results of nonsurgical management in patients younger than 12 years, with 78% of patients with satisfactory results with conservative management; however, of these, only 50% (at the 2-year follow-up) were able to keep practicing the sport they previously played. Ekas et al,^70^ in a prospective study (n = 44) of conservative treatment with an active rehabilitation protocol, showed that at least 55% of patients required late reconstruction surgery (because of residual instability, functional limitation, and secondary injuries), with a 90% overall return-to-sports rate, but 66% had to restrict their sports activity.

Kocher et al^71^ reported excellent functional outcomes with a low revision rate (4.5%) and a minimal risk of growth disturbances in 44 prepubescent patients (Tanner 1 and 2) followed up for a mean of 5 years, with physeal-sparing ACL reconstruction with ITB graft. Other studies have reported outcomes on this physeal-sparing “Micheli” technique, using ITB for combined intra-articular and extra-articular ACL reconstruction, confirming that it is a safe and effective reconstruction technique for children with several years of growth remaining.^72^

Calvo et al^73^ conducted initial surgical management with a transphyseal technique in 27 patients. It demonstrated that all patients returned to sports, and only 11% did so at a lower level of demand. In a systematic review, Kay et al^13^ showed that patients undergoing ACL reconstruction with the all-epiphyseal technique had an overall return-to-sport rate of 91%.

## Complications

Complications described in this group of patients are rerupture, limb-length discrepancy, and angular deformities. Table 1 shows a comparative summary regarding complications.

**Table 1:** Comparative Summary Regarding Risk of Rerupture and Growth Disturbances After Anterior Cruciate Ligament Surgery Reconstruction in Skeletally Immature Patients

Author (Year)	Design (Evidence Level)	Follow-up	Cases (Male/Female)	Mean age (Range)	Surgical Technique	Failure number (%)	Growth Alteration Cases (%)
Cruz et al^66^ (2017)	Retrospective (IV)	21 mo (6-66)	103 (79/24)	12.1 (6.3-15.7)	All-epiphyseal	11 (10.7)	1 (<1)
Calvo et al^73^ (2015)	Case series (IV)	10.6 yrs (10-13)	27 (16/11)	13 (12-16)	Transphyseal	3 (11.1)	0
Pierce et al^77^ (2017)	Systematic revision	Transphyseal: 4.4 yrs (2-22) physeal-sparing: 4.7 yrs (2-15)	948 (610/338)	Transphyseal: 13.5 Physeal-sparing: 11.9	Transphyseal (n = 786) Physeal-sparing (n = 162)	Transphyseal: 48 (6.2) Physeal-sparing: 5 (3.1)	Transphyseal: 7 (1.4) Physeal-sparing: 2 (1.2)
Cordasco et al^51^ (2017)	Case series (IV)	2 yrs	23 (17/6)	12.2 (9.9-14.5)	All-epiphyseal	1 (4.3)	6 (26)
Wong et al^60^ (2019)	Meta-analysis (III)	49.6 mo (16-175)	1321 (885/436)	13 (8.75–16.4)	Transphyseal (640) Physeal-sparing (277) Partial transphyseal (141)	115 (8.7) global	55 (4.2) global
Ekas et al^70^ (2019)	Case series (IV)	8 yrs (5-11)	46 (12/34)	11 (7–13)	Transphyseal	1 (4.2)	0
Patel et al^76^ (2019)	Case revision (III)	26.5 mo (±5.2)	690	All-epiphyseal (12.1 ± 1.8) Transphyseal (15.8 ± 1.9)	All-epiphyseal (133) Transphyseal (557)	All-epiphyseal 13 (9.8) Transphyseal 58 (10.4)	Not reported
Wong et al^78^ (2019)	Review	47.7 mo	478 (387/91)	Over-the-top (12.3) All-epiphyseal (11.6)	Over-the-top (298) All-epiphyseal (178)	Over-the-top 9 (3) All-epiphyseal 13 (7.3)	Over-the-top 3 (1) All-epiphyseal 6 (3.4)

Of these, the most important and most frequent is rerupture, which can occur in up to 19% of cases,^15^ regardless of the techniques used. This high rerupture rate is the main concern regarding ACL reconstruction surgery for this age group. Wiggins et al^74^ in a systematic review and meta-analysis concludes that the risk of reinjury in young athletes was 23% when returning to high-risk sports. In addition, approximately one in six of the pediatric patients who had an anterior cruciate ligament reconstruction will have another surgery within 3 years from the surgery, including contralateral ACL rupture as a cause.^75^ In the transphyseal technique, there is a risk of rerupture of <25%,^76,77^ and new meniscal lesions are observed in 15.3% of cases.^73^

Growth alterations are described as generally low, <1%.^77^ The most common angular deformation is valgus deviation, mainly when transphyseal techniques were used.^60^ Reconstruction with physeal-sparing techniques presents a risk of rerupture of <15%,^66,76-78^ growth alterations secondary to physeal arrest (including limb-length discrepancy and angular deformities) < 6% in the all-epiphyseal techniques,^66,78^ and reports of <1% in the over-the-top technique.^78^

In a systematic review that includes studies from 1986 to 2015, with a total of 21 studies reporting 39 patients, 29 cases of limb-length discrepancy, and 16 cases of angular deformity were analyzed. For limb-length discrepancy, limb overgrowth accounted for 62% of cases. Physeal-sparing techniques were conducted in 25% of the cases of angular malformation and in 47% cases of limb-length discrepancy.^79^ This shows that current understanding of the etiology of this problem is limited.

## Authors' Preferred Treatment

To date, there is insufficient evidence to recommend one treatment technique over another in children. Surgeons' preferences vary from strict clinical follow-up looking for symptomatic functional instability after conducting a conservative rehabilitation protocol to ACL reconstruction delayed until skeletal maturity.

However, it is advisable to comply with certain principles to avoid complications in this age group. Evidence promotes surgical treatment in patients who maintain subjective instability that restricts their activities or risk repeated failures to avoid additional joint damage. Always estimate the remaining growth potential using the chronological age, bone age, sex of the patient, and the tables previously mentioned. If transphyseal techniques are to be considered in patients with >1 year of remaining growth, central, vertical tunnels and soft-tissue grafts that cross the physis to resect a physeal volume <7% to 9% should be used and the fixations should be placed far away from the physis.

**Figure 8:**
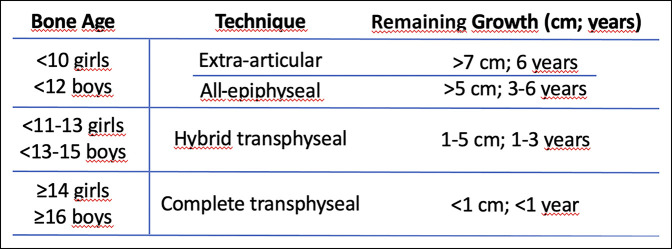
Chart showing the therapeutic treatment algorithm preferred by the authors for the choice of the anterior cruciate ligament reconstruction technique in patients with immature skeleton.

Our preferred treatment is based on remnant growth. In those patients with more than 6 years of growth potential, we prefer over-the-top reconstruction with ITB. For those patients with 3 to 6 years of remaining growth, all-epiphyseal anatomical techniques should be used. When there is between 1 and 3 years of remaining growth, hybrid (all-epiphyseal in the femur and transphyseal in the tibia) reconstruction is preferred. For those skeletally mature or with <1 year of growth remaining, usual anatomical reconstruction techniques should be used (Figure 8).

Postoperatively, we strictly followed up with serial lower extremity radiographs until the end of growth to detect physeal arrests early. We emphasize to parents and patients the importance of a rigorous and high-quality rehabilitation, and we do not authorize return to sports before 9 to 10 months after reconstruction; adequate muscle balance is also confirmed by an isokinetic test. In addition, we incorporate lesion-prevention programs during reinstatement.

## Prevention

ACL rupture is a devastating injury for young athletes, representing a notable burden for patients and their families for rehabilitation, time out of sports, costs, and future sequelae. ACL injury prevention programs have gained increased attention during the past few years and are one of the key points where trainers, therapists, and orthopaedic surgeons should focus their efforts. Prevention programs are a combination of plyometrics, strength, technique, and balancing exercises, among other neuromuscular training, aimed to reduce ACL injury rates. The goal of prevention programs is that athletes adopt safer movement patterns that reduce the risk of injury during high-risk sports, obtaining adequate control and neuromuscular positioning of the knee, avoiding dynamic valgus, and achieving knee stability during competitive sport.^27,70^ Therefore, the athlete's biomechanical movement patterns are a key modifiable risk factor to prevent ACL injuries. High-level evidence summarized in a meta-analysis by Webster et al^80^ shows conclusive evidence that ACL injury prevention programs reduce the risk of ACL injuries by half in all athletes, and the effect is even bigger in female athletes reaching a two-thirds reduction.

Risk mitigation programs are cost-effective and simple to implement because they require little or no special equipment, and they are conducted as part of regular team training or physical education class, 2 to 3 times a week. Nevertheless, adherence to these programs is difficult to obtain in young athletes and mandatory to achieve a sustained risk reduction.^81^ If these programs are implemented early in the athletic development process, it will give athletes a better chance of having adequate strategies for sudden movements and less risk of injury. The “FIFA 11 + for Kids” is a validated tool for the pediatric population that has shown to reduce global recreational and subelite soccer injuries by 39% and specifically knee injuries by 48%.^82^ In many cases, training programs are not applied early enough, and it is during postsurgical rehabilitation the time to implement them, which will contribute to reducing the incidence of reinjuries in the high-risk population.
